# Long-term Effectiveness of Maxillary and Mandibular Bonded Orthodontic Retainers

**DOI:** 10.3290/j.ohpd.a44939

**Published:** 2020-07-24

**Authors:** Katharina E. Kocher, Meret C. Gebistorf, Nikolaos Pandis, Piotr S. Fudalej, Christos Katsaros

**Affiliations:** a Orthodontist in Private Practice, Bern, Switzerland. Performed all measurements of the plaster models at the three timepoints, analysed and interpreted all data, was substantially involved in writing the manuscript, read and approved the final manuscript.; b Orthodontist in Private Practice, Aarau, Switzerland. Responsible for the ethics approval of the study, organised the recall appointments, measured plaster models to assess inter-rater reliability, co-wrote Materials and Methods section, read and approved the final manuscript.; c Associate Professor, Medical Faculty, School of Dental Medicine, Department of Orthodontics and Dentofacial Orthopedics, University of Bern, Bern, Switzerland. Statistical analysis and was substantially involved in the manuscript writing and revision, read and approved the final manuscript.; d Professor, Institute of Dentistry and Oral Sciences, Palacky University Olomouc, Olomouc, Czech Republic; Medical Faculty, Department of Orthodontics, Jagiellonian Universits, Kraków, Poland; Adjunct Professor, Medical Faculty, School of Dental Medicine, Department of Orthodontics and Dentofacial Orthopedics, University of Bern, Bern, Switzerland. Supervised implementation of trial design, data acquisition and manuscript revision, read and approved the final manuscript.; e Professor and Chair, Medical Faculty, School of Dental Medicine, Department of Orthodontics and Dentofacial Orthopedics, University of Bern, Bern, Switzerland. Supervised implementation of trial design, data acquisition and manuscript revision, read and approved the final manuscript.

**Keywords:** adverse effects, effectiveness, efficiency, failures, fixed retainers, irregularity index, long-term retention, retention

## Abstract

**Purpose:**

This retrospective cohort study investigated the long-term effectiveness of one type of maxillary and 2 types of mandibular fixed lingual retainers.

**Materials and Methods:**

Eighty orthodontic patients in retention for 10-15 years were included. Irregularity index, intercanine width, overjet (OJ) and overbite (OB) were measured on plaster models at 3 occasions: (T1) pre-treatment, (T2) post-treatment and (T3) 10-15 years post-treatment. Analyses assessed the effect of the retainer type and time on mandibular irregularity, intercanine width and retainer failure.

**Results:**

In the mandible, the irregularity index increased (0.43 mm) between T2 and T3 for the 0.027” β-titanium (TMA) retainers bonded to canines only while it was stable (-0.02 mm) for the 0.016” x 0.022” braided stainless steel retainers (SS6) bonded to all six anterior teeth. The intercanine width was relatively stable in both groups during the entire observation period. In the maxilla, the irregularity index was stable between T2 and T3 (+0.07 mm). The intercanine width increased (+2.02 mm) during treatment T1-T2 and was stable (-0.02 mm) in the retention phase T2 to T3.

**Conclusions:**

In the mandible, SS6 retainers were slightly more effective in maintaining alignment compared to the TMA retainers. In the maxilla, the SS4 retainers without canine extensions were effective in maintaining alignment. All retainers were effective in maintaining the intercanine width.

Orthodontic relapse is unpredictable.^21-24^ It has been hypothesised that periodontal,^31^ occlusal,^10,15^ and soft tissue factors^30^ play a role, however without clear association between specific parameters and relapse.^20,21^ It is common practice to implement a retention plan following the completion of orthodontic therapy in order to prevent relapse. A variety of retention protocols have been used over the years including removable, fixed or a combination of those appliances.^4-6,18,25,27,29,32,35^ More recently, vacuum-formed retainers have seen increased popularity due to associated comfort and aesthetics compared to the original removable retainers.^9,13,25^ Fixed retainers using different metal compositions, shapes and dimensions are very popular especially for the mandible. They do not have an impact on aesthetics and are less demanding in terms of long-term cooperation.^14^ A recent Cochrane review found no clear evidence of greater stability with vacuum formed retainers worn full-time vs part-time with a paucity of high-quality evidence on recommendations for the stability of orthodontic results.^24^

Long-term retention imposes further responsibility on the practitioner in terms of explaining the consequences of this approach. Patients must adhere to advice in order to maintain the treatment results and prevent any unexpected adverse events.^16,28^

Given the long retention periods, prospective studies are likely to have statistically significant and uneven losses to follow-up, which can bias results. In addition, the plethora of the existing retention protocols makes it difficult for all of them to be tested in an RCT setting. The small number of RCTs in the area usually include short follow-up periods and therefore it is common to gather evidence on long-term retention from observational studies, from which results can be used to develop more targeted clinical trials.^4,24^

To our knowledge, a fixed retention protocol was assessed only in one study with an observation duration >10 years.^8^ No retainers made from β-titanium (TMA) wire bonded only to mandibular canines have ever been assessed long-term. The goal of this study was therefore to assess the effectiveness of a maxillary retainer and two different mandibular retainers to maintain anterior alignment 10 and 15 years after treatment.

## Materials and Methods

This retrospective cohort study is a follow-up study of Kocher et al.^17^ The longitudinal sample reported by Gebistorf et al^12^ was used with slightly modified inclusion and exclusion criteria. The study was approved by the Ethics Committee of Bern, Northwest and Central Switzerland (EKNZ 2015-349, HVF, Kat A). Every patient signed an informed consent before inclusion in the study. The STROBE guidelines for reporting of observational studies were followed.^37^

### Participants

The sample included patients from a private orthodontic practice in Switzerland. In this practice, it was routine to keep pre-treatment (T1) and post-treatment records (T2) for at least 10 years after the last retainer check-up visit. The last orthodontic visit was usually performed between 1 and 4 years post-treatment before patients were referred to their private dentist for future assessment of the bonded retainers. A 2-phase treatment for growth modification with a removable appliance, extractions, interproximal enamel reduction or surgery was applied if necessary, according to the orthodontic treatment protocol. No circumferential supracrestal fiberotomy was performed.

No prior sample size calculation was performed; however, all eligible patients were considered if they met the inclusion criteria: (A) treated with fixed appliances; (B) treated by the same orthodontist; (C) maxillary and mandibular retainers bonded immediately after completion of active orthodontic treatment and (D) non-syndromic patients. No age restriction was applied. For qualified patients, a recall appointment was scheduled which involved (I) a clinical examination; (II) taking photos and (III) alginate impressions. The response rate for the recall appointment was 70.7%. Additional exclusion criteria were applied after the recall appointment: (A) orthodontic retreatment; (B) post-treatment appointment (i.e. retention time, T3) less than 10 or more than 15 years ago; (C) retention phase with no/other mandibular lingual retainer than (a) 0.016” x0.022” braided SS bonded to all 6 mandibular anterior teeth (SS6) or (b) 0.027” round β-titanium bonded to canines only (TMA) and maxillary retainer 0.016” x 0.022” braided SS bonded to 4 (SS4) or all 6 (SS6) anterior maxillary teeth; (D) different/modified mandibular or maxillary retainer in situ at T2 and T3 without information and (E) mandibular or maxillary retainer removed for prosthetic restorations or modified. Eighty patients were finally included for the present study. Details of patient selection have been presented previously.^17^ Eight additional patients were excluded because of missing or broken models during transport.

### Retention Protocol

**Fig 1:**
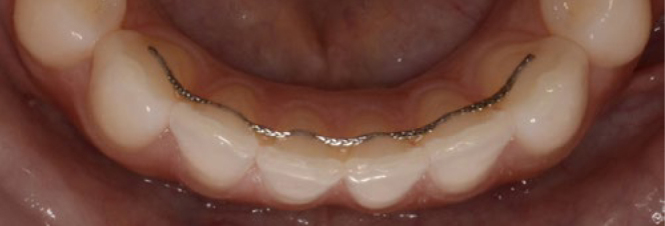
0.016” x 0.022” eight-strand braided SS wire (D-Rect. ORMCO) bonded to all 6 mandibular anterior teeth at T3 (SS6).

**Fig 2:**
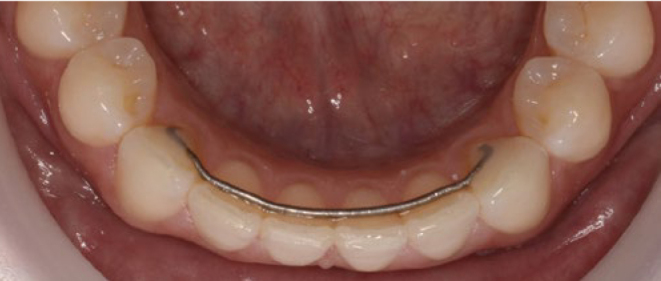
0.027” round β-titanium wire bonded to lower canines only (ORMCO) at T3 (TMA).

Two types of mandibular retainers were used: (A) 0.016” x 0.022” eight-strand braided SS wire (D-Rect. ORMCO; Orange, CA, USA) bonded to all 6 lower anterior teeth (incisors and canines) (Fig 1) and (B) 0.027” round β-titanium wire bonded to canines only (ORMCO) (Fig 2).

The TMA wires were sandblasted at the bonding sites. The choice of retainer was made by the orthodontist according to the oral hygiene status and the initial amount of crowding. In patients with good hygiene and/or significant initial crowding, a retainer bonded to all 6 lower anterior teeth was chosen. In patients with poor oral hygiene and/or little initial irregularity a TMA retainer bonded only to the canines was administered. The initial amount of irregularity was considered more important than the current level of oral hygiene for the choice of the type of retainer; thus, patients with high initial malalignment and poor oral hygiene were retained with a SS6 retainer.

**Fig 3:**
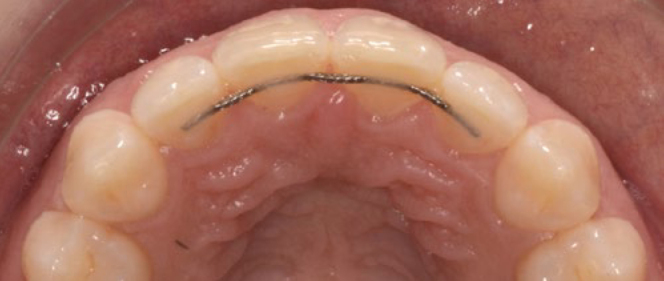
0.016” x 0.022” braided SS wire (D-Rect. ORMCO) bonded to all 4 maxillary incisors at T3 (SS4).

**Fig 4:**
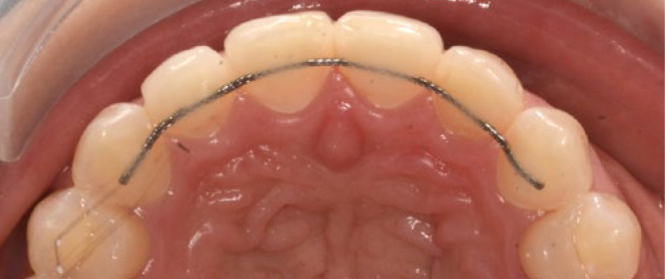
0.016” x 0.022” braided SS wire (D-Rect. ORMCO) bonded to all 6 maxillary anterior teeth at T3 (SS6).

The standard retainer in the maxilla was 0.016” x 0.022” braided SS wire (D-Rect., ORMCO; Orange, CA, USA) bonded to all 4 incisors (Fig 3). For 6 patients, the wire was extended to the canines due to severe pre-treatment displacement (T1) (Fig 4).

All retainers were placed according to a standardised procedure by the same orthodontist. The tooth surfaces were cleaned with a low-speed handpiece using a rubber cup with non-fluoridated pumice and sandblasted. Thereafter, the enamel was etched with 37% phosphoric acid gel for 30 s, washed and air dried. The bonding agent (Ortho Solo, ORMCO) was applied and light cured. The retainers were manually placed in the correct position on the teeth, stabilised with a high viscosity composite (Charisma, Kulzer; Hanau, Germany) and then covered with a thin layer of flowable composite (Flow Tain, ORTHOBY; Rudolfstetten, Switzerland).

### Data Collection

**Fig 5:**
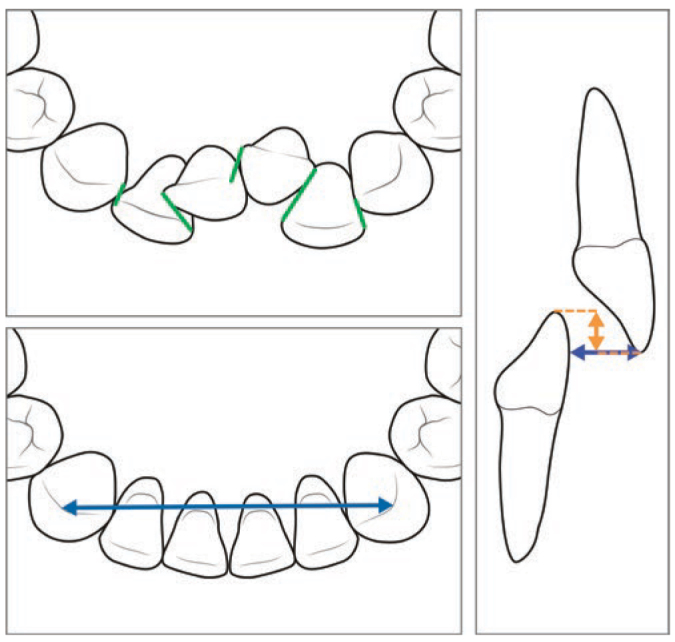
Schematic representation of the measured variables: irregularity index, intercanine width, overbite and overjet.

The primary outcome for the present study was retainer effectiveness expressed by Little’s irregularity index (LII).^19^ Secondary outcomes were stability of the intercanine width and intermaxillary changes expressed by overjet and overbite. Dental casts were measured on three occasions: pre-treatment (T1), post-treatment (T2) and 10-15 years post-treatment (T3) by one examiner (KK), in line with a previous report^11^ (Fig 5).

- Irregularity index: The summed displacement of the anatomic contact points of the maxillary and mandibular incisors and canines not including the distal contact points of the canines to premolars.
- Intercanine width: The distance between the canine cusp tips. In case of abrasion of a tip, an estimation of the middle of the surface was made.
- Overbite: The overlap of maxillary to mandibular central incisors.
- Overjet: The distance parallel to the occlusal plane from the incisal edge of the most labial maxillary incisor to the opposing mandibular central incisor.

T1 often coincides with the transition period from primary to permanent dentition. The irregularity index and the intercanine width were not measured in case of missing canines at T1 (mandible 11 out of 80 patients, maxilla 15 out of 75 patients). No hypothetical position of the missing canines was assumed.

The measurements were made with an electronic digital caliper (Art.Nr. H-59112, FINO; Mangelsfeld, Germany) with a precision of 0.01 mm. All three sets of dental casts (T1, T2, T3) from one patient were measured sequentially. The dental casts where encoded with a patient ID. There was no blinding of the investigator regarding the type of retainer. All measurements were completed within 4 weeks.

### Method Error

The casts of 25 patients (total: 75 casts) were randomly selected and remeasured by the same examiner (KK) on a second occasion after an interval of 4 weeks to asses intra-rater reliability. These casts were also measured by a second examiner (MG) to appraise inter-rater reliability. Two calibration sessions were held before the measurement sessions. In the first calibration session, the two examiners discussed the measurement method on one randomly chosen cast. Possible difficulties were discussed before 3 sets of casts, not included later in the study, were measured separately by both examiners. Any discrepancies of measured values were then discussed until a consensus was reached.

Demographic data such as gender, age, and treatment duration were obtained from the clinical records. The history of retainer failure was obtained from the data of a previous study.^17^ Periodontal outcomes were not considered in the present study. Adverse effects including torque or movement of teeth with intact bonded wires and wire failures were assessed in a previous paper.^17^

### Statistical Analysis

Baseline characteristics were calculated for the 2 mandibular and the 2 maxillary retainer groups. It was then decided to exclude the 3 patients with maxillary retainers bonded to 6 anterior teeth from further analysis because of the very small sample size. Descriptive statistics were computed for the LII and intra- and intermaxillary linear dimensions at T1, T2, and T3. The retainers were compared between them and over time. A mixed model was additionally fitted to assess the effect of retainer type and time as well as their interaction on mandibular LII. Linear regression was implemented separately for the mandible and the maxilla to examine potential associations between the LII at T3 and the type of retainer, history of retainer failure and age at T3. Intra- and inter-rater reliability were calculated with the intraclass correlation coefficient (ICC). Statistical significance for all tests was set at 0.05. All analyses were performed using Stata 15.1 statistical software (Statacorp; College Station, TX, USA).

## Results

Patients’ demographic characteristics according to the type of mandibular and maxillary retainer across time are shown in Tables 1a and 1b.

**Table 1a:** Baseline characteristics per retainer type and timepoint for the mandible

		0.016” x 0.022” braided SS			0.027” TMA		
		T1 (N = 43) mean (SD) or (%)	T2 (N= 43) mean (SD) or (%)	T3 (N= 43) mean (SD) or (%)	T1 (N = 37) mean (SD) or (%)	T2 (N = 37) mean (SD) or (%)	T3 (N = 37) mean (SD) or (%)
Age (y)		12.4 (3.0)	15.7 (3.0)	28.1 (3.0)	12.1 (1.6)	14.9 (1.7)	28.1 (1.9)
Gender	male	11 (25.6)	11 (25.6)	11 (25.6)	12 (32.4)	12 (32.4)	12 (32.4)
	female	32 (74.4)	32 (74.4)	32 (74.4)	25 (67.6)	25 (67.6)	25 (67.6)
Duration (y)	treatment (T1–T2)			3.1 (1.5)			2.8 (1.0)
	retention (T2–T3)			12.5 (1.2)			13.2 (1.1)
Type of maxillary retainer	4 incisors	–	–	40 (93.0)	–	–	35 (94.6)
	6 anterior teeth	–	–	3 (7.0)	–	–	2 (5.4)

**Table 1b:** Baseline characteristics per retainer type and timepoint for the maxilla

		0.016” x 0.022” braided SS 4 incisors			0.016” x 0.022” braided SS 6 incisors		
		T1 (N = 75) mean (SD) or (%)	T2 (N= 75) mean (SD) or (%)	T3 (N= 75) mean (SD) or (%)	T1 (N = 5) mean (SD) or (%)	T2 (N = 5) mean (SD) or (%)	T3 (N = 5) mean (SD) or (%)
Age (y)		12.0 (2.2)	14.9 (2.2)	27.8 (2.4)	13.8 (3.8)	18.2 (3.0)	30.3 (2.5)
Gender	male	22 (29.3)	22 (29.3)	22 (29.3)	1 (20.0)	1 (20.0)	1 (20.0)
	female	53 (70.7)	53 (70.7)	53 (70.7)	4 (80.0)	4 (80.0)	4 (80.0)
Duration (y)	treatment (T1–T2)			2.8 (1.2)			4.4 (1.9)
	retention (T2–T3)			12.8 (1.2)			12.1 (1.2)
Type of mandibular retainer	TMA	–	–	34 (45.3)	–	–	3 (60%)
	braided SS	–	–	41 (54.7)	–	–	2 (40%)

Patients with a maxillary SS6 retainer were not further analysed because of the very small sample size. The intra- and inter-rater reliability of the outcome measures was high for both jaws, with all outcomes ranging from 0.866 to 0.997.

### Mandible T1-T3 (Table 2)

**Table 2:** Means and standard deviations for irregularity index, intercanine width, overjet and overbite per retainer type and timepoint in the mandible

Mandible	ss6			tma		
	T1	T2	T3	T1	T2	T3
Irregularity Index						
n	35*	43	43	34*	37	37
mean (SD)	6.32 (3.55)	1.01 (0.53)	0.99 (0.69)	3.25 (1.81)	0.97 (0.45)	1.40 (0.63)
Intercanine width						
n	35*	43	43	34*	37	37
mean (SD)	26.37 (1.80)	26.83 (1.27)	26.66 (1.25)	26.46 (2.07)	26.18 (1.83)	26.47 (1.94)
Overjet						
n	43	43	43	37	37	37
mean (SD)	4.26 (2.95)	2.34 (0.51)	2.15 (0.75)	4.89 (2.28)	2.13 (0.52)	2.37 (2.46)
Overbite						
n	43	43	43	37	37	37
mean (SD)	3.78 (1.54)	2.13 (0.41)	1.99 (0.71)	3.86 (1.66)	1.86 (0.62)	1.64 (2.14)

n=sample size, SD=standard deviation, * value absent for some patients because of missing canines (transition from primary to permanent dentition).

**Fig 6:**
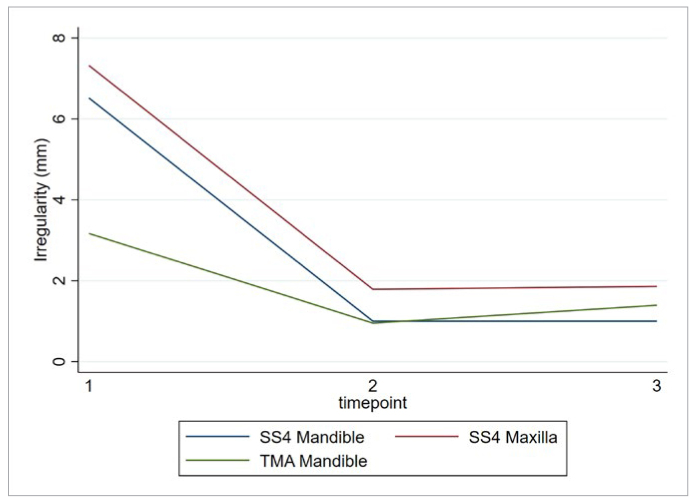
Mean irregularity index per type of retainer and time.

At the start of orthodontic treatment (T1), the mean LII was 6.32 mm (SD 3.55) in the SS6 group and 3.25 mm (SD 1.81) in the TMA group. After treatment (T2), LII was around 1 mm for both groups and a minimal increase (+0.43 mm) was recorded in the LII during the retention period (T2-T3) for the TMA group but not for the SS6 group. After 10-15 years of retention, the mean LII in the SS group was 0.99 (SD 0.69) mm and in the TMA group 1.4 mm (SD 0.63) (Fig 6).

**Fig 7:**
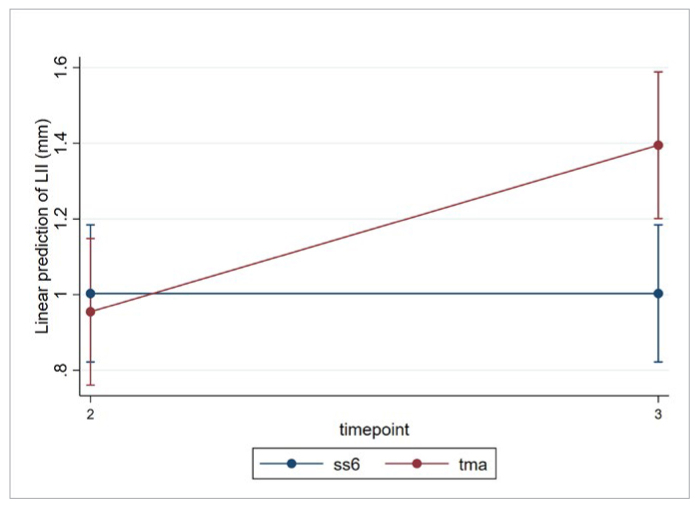
Predicted LII values in mm for SS6 and TMA retainers at T2 and T3.

In both groups, the intercanine width was relatively stable and varied only within a minimal range (0.46 mm SS6, 0.19 mm TMA) over all three timepoints. Tooth attrition could have introduced a small measurement error for the intercanine width.

### Maxilla T1-T3 (Table 3)

**Table 3:** Means and standard deviations for irregularity index and intercanine width

Maxilla	ss4		
	T1	T2	T3
Irregularity Index			
n	60*	75	75
mean (SD)	7.32 (3.59)	1.79 (0.68)	1.86 (1.10)
Intercanine width			
n	60*	75	75
mean (SD)	33.30 (2.73)	35.32 (1.78)	35.3 (2.08)

n=sample size, SD=standard deviation, * value absent for some patients because of missing canines (transition from primary to permanent dentition).

Similar to the mandible, maxillary LII decreased for patients with a SS4 retainer during treatment (T1-T2) from 7.32 mm (SD 3.59) to 1.79 mm (SD 0.68) and increased minimally (+0.07 mm) in the retention time. At T3, mean LII was higher in the maxilla (1.86 mm, SD 1.1) than in the mandible (SS6 0.99 mm, SD 0.69 vs TMA 1.4 mm, SD 0.63) (Fig 6).

The intercanine width increased on average 2.02 mm during treatment and changed only minimally in the retention period T2-T3 (-0.02 mm).

### Interactions

A statistically significant interaction between the mandibular type of retainer and time was observed for the LII (Table 4), suggesting that the difference for those outcomes between the wires changes over time. However, those changes were of little clinical importance.

**Table 4:** Mixed effects multiple linear regression for the effect of retainer type, time and their interaction on irregularity

Irregularity Index		coefficient (95% CI)	P > |z|
Retainer	SS6	Reference	–
	TMA	-0.048 (-0.31, 0.22)	0.720
Timepoint	T2	Reference	–
	T3	0.000 (-0.20, 0.20)	1.000
Retainer#timepoint	SS6	Reference	–
	TMA#3	0.440 (0.15, 0.73)	0.003

The type of retainer (SS6 vs TMA) was statistically significantly associated with the LII at T3 in the mandible (Table 5). No association was found for history of retainer failure and patient age at T3 in the mandible but age was statistically significant for the maxilla (Table 5).

**Table 5:** Multiple linear regression analysis for the effect of type of retainer, failure and age at T3 on the irregularity index at T3 in the mandible and the maxilla

Predictor		Mandible		Maxilla	
		Coefficient (95% CI)	p> |z|	Coefficient (95% CI)	p> |z|
Type of retainer	SS6	reference	–	–	–
	TMA	0.41 (0.09, 0.73)	0.014	–	–
Failure	no	reference	–	reference	–
	yes	0.13 (-0.20, 0.45)	0.441	-0.16 (-0.57, 0.26)	0.45
Age at T3 (per unit = 1 year)		-0.01 (-0.06, 0.08)	0.759	0.11 (-0.18,| -0.03)	0.006

## Discussion

We aimed to assess the occlusal stability over 10 to 15 years after treatment of a maxillary retainer (0.016″ x 0.022″ braided stainless steel (SS) wire bonded to all four anterior teeth) and two different mandibular retainers (A) 0.016″ x 0.022″ braided SS wire bonded to all six mandibular anterior teeth (incisors and canines) and (B) 0.027″ round β-titanium wire bonded to mandibular canines only. This is the first study to examine long-term stability using β-titanium (TMA) wires bonded only to mandibular canines. No other study assessed a fixed retention protocol with an observation period over 10 years, which included a clinical examination, photographs, and dental casts.

Assessment of the intra- and inter-examiner reliability was better at T1 compared to T2 and T3 for the irregularity index, probably due to the smaller values at T2 and T3. Irregularity, as expected, varied among patients and decreased substantially with treatment.

We found evidence of slightly higher irregularity at T3 for the TMA wire. This can be possibly explained by the fact that only the canines were bonded. The differences were of little clinical importance. Our results are in agreement with previous medium and long term studies.^2,7,8,26,33,34^ Analysis of the effect of retainer type adjusted for age and failures during the post-treatment follow-up period confirmed previous results, and suggested that retainer failure and age were not statistically significant predictors of post-treatment irregularity in the mandible, whereas age was a significant predictor in the maxilla. However, this may be influenced by the setting, repair and any hiatus between fracture and repair. There is evidence in the literature that there is no difference between removable and fixed retainers in terms of post retention irregularity.^24,36^ Abdulraheem et al^1^ suggested that at least 25% of the postretention changes, not predictable in direction, may be attributed to natural growth and not to postretention relapse. It would be difficult, however, to dissociate those 2 events, and from the patient’s perspective, it does not change the need for long-term retention.

In this study, irregularity in the maxilla was greater than in the mandible at all timepoints. This could be attributed to the difference in shape and size of the maxillary canines and incisors compared to their mandibular counterparts. Again, changes during the post-treatment period were slight and not clinically relevant. Previous retainer failure was not associated with increased irregularity at T3, but age was a statistically significant predictor. In a study with a 7-year follow-up, Steinnes et al^36^ concluded that, unlike in the mandible, changes in the maxilla did not differ between patients still in retention at the end of follow-up vs patients who had lost their retainer.

In the present study, overall irregularity was more variable in the maxilla. TMA retainers showed the least variability of irregularity values because only patients with low mandibular crowding received this type of retainer, while patients with severe crowding received an SS6 retainer.

There remains a lack of long-term, high-quality studies assessing orthodontic stability using fixed retainers. This should be considered as a research priority, given the importance of retention in the stability of the orthodontic treatment.^2^ Long-term retention poses a potential threat to the periodontal tissues as this has been also highlighted in a recent RCT.^2^ In terms of periodontal outcomes, there is evidence^2,3^ of no statistically significant differences between mandibular stainless-steel fixed retainers bonded to the anterior teeth and canines only at 1 and 3 years. Mandibular Hawley retainers in comparison to mandibular stainless-steel fixed retainers, also showed no statistically significant differences at the 3-year follow-up in terms of periodontal outcomes. However, in this study periodontal effects were not considered.

### Negative Effects

No undesired changes of the root torque or important negative effects were observed in any patient. For details see Kocher et al.^17^

### Limitations

The retrospective nature of the study makes it more vulnerable to bias.

The fact that this study was carried out in a single office and that the choice of mandibular retainer type and bonding procedures were always carried out by the same clinician makes the results less generalisable.

The response rate (70.7%) could have biased our findings. It is impossible to determine the direction and magnitude of the influence of losses on our results, i.e. whether participants who dropped out from the study had more, comparable, or fewer changes than those remaining. Our sample may have been subject to self-selection bias because, for example, patients pleased with the treatment result and better stability of the alignment might be more willing to respond to a follow-up examination notification.

Measurement of the intercanine or intermolar width can be difficult due to attrition, which is more likely to occur at later timepoints in life (T3 > T2 > T1). The LII tends to underestimate the irregularity of the alignment because the distal facets of the canines are not included. Other possible confounding factors cannot be fully excluded in this study.

## Conclusions

Within the limitations of this retrospective study, we conclude that:

- Mandibular 0.016” x 0.022” braided SS retainers bonded to all 6 mandibular anterior teeth are more effective in maintaining anterior alignment compared to 0.027” round β-titanium retainers bonded only to the canines.
- Both mandibular retainers maintain intercanine width.
- In the maxilla, 0.016” x 0.022” braided SS retainers maintain anterior alignment.
- Maxillary intercanine width seems to be stable without extension of the retainer to the canines.
